# TrendyGenes, a computational pipeline for the detection of literature trends in academia and drug discovery

**DOI:** 10.1038/s41598-021-94897-9

**Published:** 2021-08-03

**Authors:** Guillermo Serrano Nájera, David Narganes Carlón, Daniel J. Crowther

**Affiliations:** 1 Division of Cell and Developmental Biology, School of Life Sciences, University of Dundee, Dundee, DD1 5EH UK; 2 Division of Population Health and Genomics, Ninewells Hospital, School of Medicine, University of Dundee, Dundee, DD1 9SY UK; 3 Exscientia Ltd, Dundee One, River Court, 5 West Victoria Dock Road, Dundee, DD1 3JT UK

**Keywords:** Target identification, Data mining, Literature mining

## Abstract

Target identification and prioritisation are prominent first steps in modern drug discovery. Traditionally, individual scientists have used their expertise to manually interpret scientific literature and prioritise opportunities. However, increasing publication rates and the wider routine coverage of human genes by omic-scale research make it difficult to maintain meaningful overviews from which to identify promising new trends. Here we propose an automated yet flexible pipeline that identifies trends in the scientific corpus which align with the specific interests of a researcher and facilitate an initial prioritisation of opportunities. Using a procedure based on co-citation networks and machine learning, genes and diseases are first parsed from PubMed articles using a novel named entity recognition system together with publication date and supporting information. Then recurrent neural networks are trained to predict the publication dynamics of all human genes. For a user-defined therapeutic focus, genes generating more publications or citations are identified as high-interest targets. We also used topic detection routines to help understand why a gene is trendy and implement a system to propose the most prominent review articles for a potential target. This TrendyGenes pipeline detects emerging targets and pathways and provides a new way to explore the literature for individual researchers, pharmaceutical companies and funding agencies.

## Introduction

Pharmaceutical companies are actively looking for ways to reduce their attrition rates, the time taken for drug development, and the associated development costs^1–4^. One approach being explored to address this productivity challenge is the exploitation of big biomedical data sets through machine learning^5,6^. Evidence is emerging that machine learning can be used to speed-up and reduce the costs in all stages in drug discovery^5,6^: drug repurposing^7,8^, clinical trials^9,10^, de-novo drug design^11–20^, and target-disease associations^21–25^. However, target identification and prioritisation remain the first step for the majority of drug discovery programmes^25–28^. Only 10% of drug targets progress through clinical trials^28–30^ and this success rate appears lower for novel targets^30–32^. Historically, target identification has been broadly carried out on a case-by-case basis, based on the scientific interpretation of the available literature. However, thousands of peer-reviewed articles are published every day without taking into account pre-prints, patent data, and clinical trial reports^33^. PubMed alone contains more than 30 million publications as of 2020, and the scientific output doubles every nine years^34^, creating a corpus of "undiscovered public knowledge"^35^. Thus, there is a high demand for machine learning and other computational methods to exploit the current knowledge and facilitate the maintenance of an overview of this overwhelming literature volume. The development of (i) alert systems to identify and rank emerging targets at genomic-scale and (ii) recommendation systems to prioritise detailed reading of scientific reviews is of importance for both pharmaceutical companies and the whole scientific community^25,27,36^.

One of the most significant obstacles for the automatic analysis of biomedical literature is the use of non-redundant alternative gene synonyms, symbols, and acronyms from competing sources that can have other meanings in different areas of research^37^. Therefore, it is imperative to disambiguate biomedical entities in the scientific literature at the outset. There have been several attempts in this line of research^21–24,37–46^. However, these attempts do not unambiguously map gene and disease entities in scientific literature to controlled ontologies nor do they define an ambiguity measure for gene and disease synonyms. Although there have been multiple attempts about trend detection and burst term detection^48,49^ and more concretely about the biomedical literature of targets and small molecules^50–52^ to our knowledge this is the first attempt to analyse emerging trends about human protein coding genes.

Here we propose a new disambiguation algorithm based on co-citation networks and natural language processing to obtain accurate publication dynamics for every coding-gene in the human genome. This time-series data was used to train recurrent neural networks (RNN) in historical data and predict the state of the literature in recent years. We identify which genes are being mentioned in the literature more than expected in order to highlight and rank potential targets. This genome scale ranking is not alone sufficient for target assessment since this will not include assessment of tractability, commercial opportunity or clinical translatability, but identification of emerging biology is a key component of novel target identification. When the actual number of published articles exceeds predictions, there may have been a paradigm shift for that particular gene. Finally, we implemented topic detection algorithms along with recommendation systems to validate trendy targets. Therefore, the aims of this paper are fourfold: (i) to unambiguously detect genes and diseases within articles with a novel named entity recogniser (ii) to generate a ranking of genes and diseases based on a novel metric that defines its trendiness, (iii) to generate an automatic pipeline to analyse why these biological entities may be trendy, and (iv) to generate a recommendation system to suggest which articles to read which maximise the information coverage in subnetworks.

## Results

### Gene annotation

**Figure 1:**
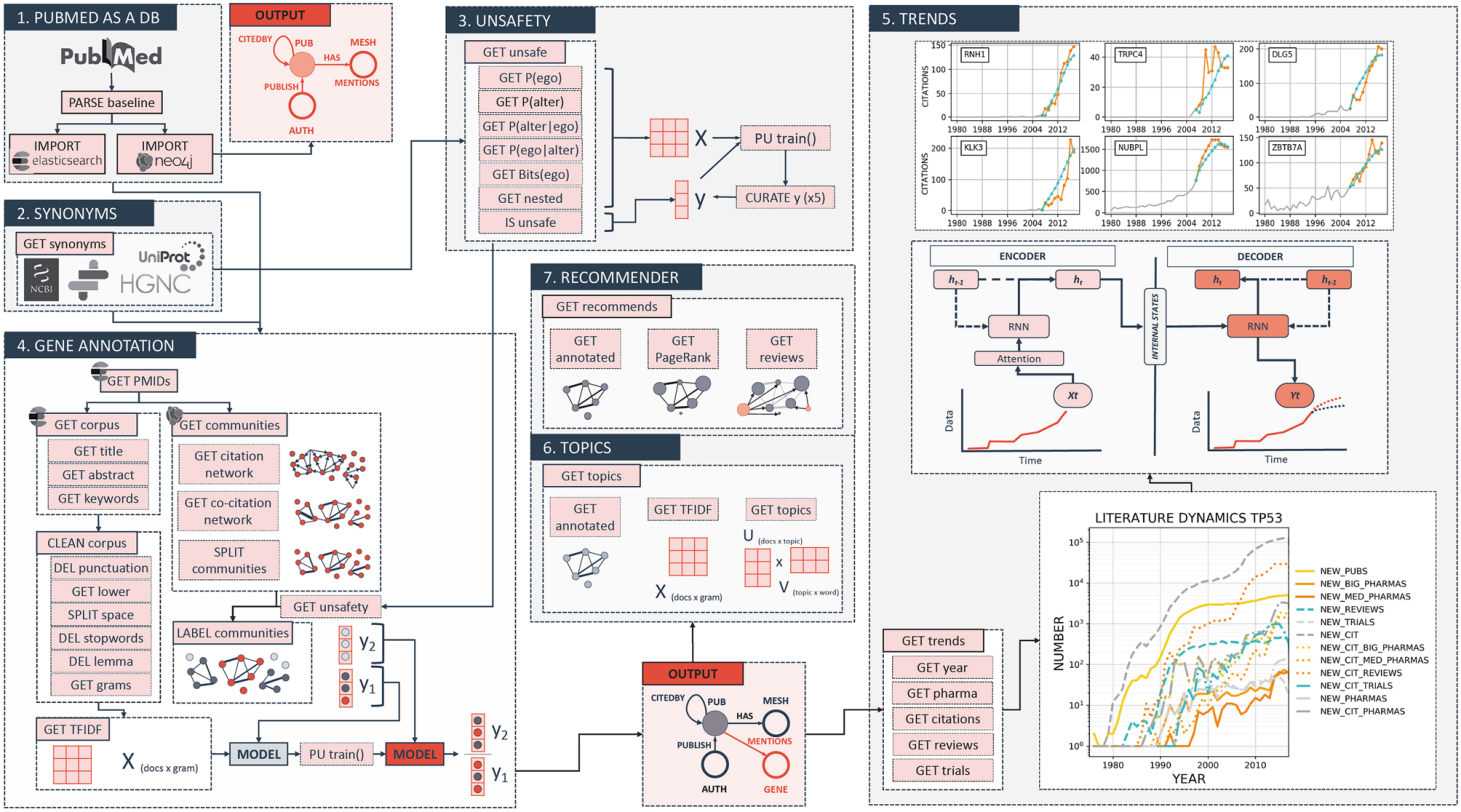
Workflow. Chart summarising the process from the downloading of the data to the detection and analysis of trends in the literature. (**A**) Creation of a graph database with the information contained in PubMed baseline 2020. (**B**) Acquisition of a comprehensive collection of human coding gene names and synonyms. (**C**) Automatic determination of potential ambiguous (unsafe) gene names. (**D**) Annotation of the graph database with unambiguous gene symbols by combining co-citation network topology and binary classifiers. (**E**) Prediction of per-gene publication trends using RNN. When a gene has significantly more publications or citations than expected by the model it is considered to be trendy. (**F**) Automatic topic detection of collections of publications. We used this algorithm to quantify the evolution of topics in trendy gene publications over time. (**G**) A review recommender system that uses information from the citation network and topic detection to recommend the most efficient set of reviews to explore the literature.

We gathered the human gene synonyms from different sources (Ensembl, UniProt, HGCN, Entrez and OpenTargets; Fig. 1B) to sample the potential publications mentioning human gene names. Human genes had around 10 synonyms on average and many of those synonyms are ambiguous (Table 1): More than 30% of gene symbols had at least one promiscuous synonym, around 10% of the gene symbols are unsafe and have at least one gene synonym in the English dictionary, and almost 50% of gene symbols had a nested synonym. Combining these problems, almost 60% of the 19,082 gene symbols have one or more of these four types of ambiguity. To determine which synonyms are potentially ambiguous (“unsafe gene synonyms”; Fig. 1C) we did feature engineering to obtain variables that characterise unsafe synonyms (e.g. longer gene names are less probable to be ambiguous; Table 2). Next, we used a positive-unlabelled bagging (PU) strategy following Mordelet et al. implementation^55^ with a random forest classifier with the engineered features to calculate the probability of a gene synonym to be “unsafe” (see Methods).

**Table 1:** Gene synonyms are ambiguous.

Type of synonym	Total counts	Percentage of the total number of synonyms
Nested	18,845	10.16
Promiscuous	11,744	6.32
English	1247	0.67
Manually discarded	58	0.03
Unsafe	24,491	13.20

Manually discarded synonyms were labelled as unsafe during the unsafe gene synonym detection in an active learning fashion (see Methods). Unsafe aggregates the data from all the other categories. Data for 19,082 gene symbols and 185,549 gene synonyms. The total counts represent the number of individual synonyms when grouped by gene symbol and gene synonym. Promiscuous synonyms are counted as many times as they act a synonym.

**Table 2:** Unsafe features.

Variable	Meaning
Total	Number of total PubMed ID candidates retrieved in ElasticSearch when querying for all gene synonyms for a given gene symbol
Contribution	The percentage of PubMed IDs that a given gene synonym contributes to the total for a particular gene symbol
Number of characters	The length of the gene synonym in characters
Bits	The sum of the bits of information of every character in a gene synonym based on the frequencies of each character in PubMed’s corpus of titles and abstracts
Number of nested	The number of other gene synonyms that contain the gene synonym. For example: “Insulin” is part of “Insulin Receptor”
Prob. of the synonym given an alternative	The conditional probability of finding the gene synonym given that an alternative synonym for the same gene symbol also appears in the text
Prob. of an alternative given the synonym	The conditional probability of finding alternative gene synonyms given that the synonym synonym appears in the text
Is gene symbol	Whether the synonym is also an accepted gene symbol

Engineered features to evaluate the probability of a given gene symbol of being ambiguous (unsafe).

To link every human gene to a subset of publications we implemented a disambiguation pipeline based on co-citation networks and machine learning (Fig. 1D). We gathered the titles, abstracts and keywords of the publications that had a match for any of the synonyms using regex with ElasticSearch (Fig. 1D). Nevertheless, this original set of publications potentially contains false positives: publications that contain an ambiguous gene synonym in their titles or abstracts, that do not refer to the gene of interest.

**Figure 2:**
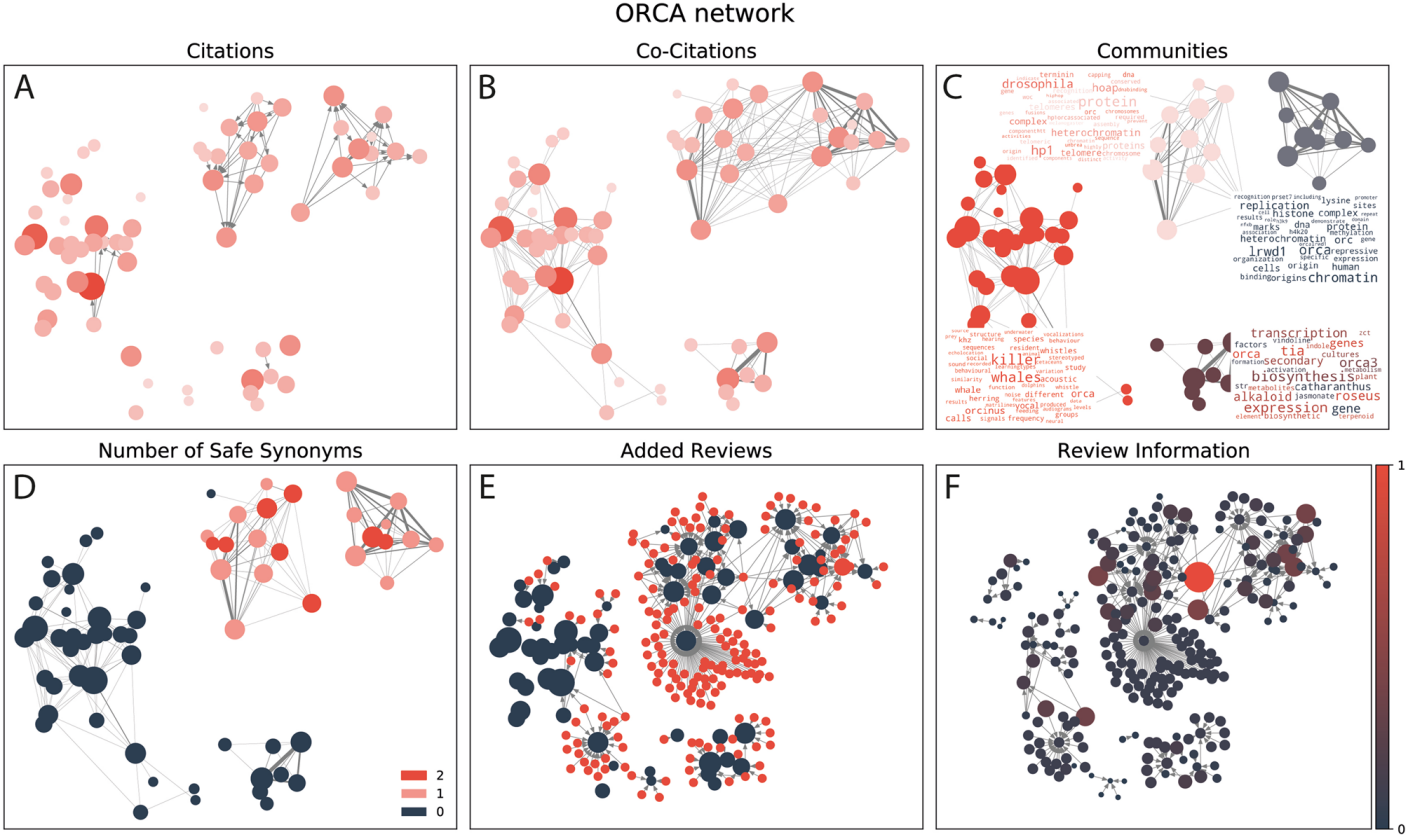
Disambiguation pipeline. **(A)** Citation network for a subset of PubMed IDs mentioning any of the gene synonyms of the gene symbol LRWD1, including ORCA. **(B)** Co-citation network of the same subset of PubMed IDs as in **(A)**. **(C)** Communities for the co-citation graph obtained after using iGraphs fast greedy algorithm: killer whale community, orca plant cluster, LRWD1 in drosophila and LRWD1 in heterochromatin. **(D)** Number of safe synonyms per PubMed ID in title or abstract in the same co-citation network. **(E)** Citation network with reviews citing any of the PubMed IDs. **(E)** Review information as defined by the recommender system scaled from 0 to 1.

We assumed that true and false positives synonyms will tend to belong to different communities of publications from different research fields. To detect these communities we used co-citation networks (Fig. 1D): a weighted graph where the weight of the edges represents the frequency of two publications being cited simultaneously (co-cited) by a third publication. When two publications are repeatedly co-cited it strongly suggests that both belong to the same field of study^56^. We used the fast greedy modulation algorithm from iGraph to determine communities in the co-citation network and distinguished communities of publications focusing on the gene of interest by detecting the presence of “safe gene synonyms” in their titles and abstracts (Fig. 1D). The process is summarised in Fig. 2.

Finally, because we only used citations from open-access publications contained in PubMed Central (PMC)^57^, 46% of the publications were disconnected in the PubMed co-citation graph. To tackle this problem, we used again the inductive bagging positive-unlabelled approach to train multiple classifiers to associate the disconnected publications with the previously computed co-citation network components (Fig. 1C) using the words, phrases and one to four n-grams, contained in titles and abstracts. All available machine classifiers in Scikit Learn were used but logistic regression was selected due to its speed to accuracy ratio (Table 3).

**Table 3:** Classifier comparison.

Classifier	Accuracy	Average precision	Brier loss	F1	Log loss	Precision	Recall	AUC	Time (s)
ETC	0.95	0.93	0.05	0.95	1.71	0.95	0.95	0.95	1.35
GPC	0.88	0.85	0.12	0.87	4.25	0.89	0.88	0.88	6.12
KNC	0.86	0.84	0.14	0.86	4.74	0.89	0.86	0.86	2.22
**LOG**	**0.93**	**0.91**	**0.07**	**0.93**	**2.36**	**0.94**	**0.93**	**0.93**	**0.54**
MLP	0.92	0.89	0.08	0.92	2.85	0.91	0.92	0.92	1.27
RDC	0.86	0.83	0.14	0.86	4.74	0.87	0.86	0.86	0.22
RFC	0.95	0.93	0.05	0.95	1.81	0.95	0.95	0.95	1.26
SVC	0.94	0.92	0.06	0.94	2.14	0.94	0.93	0.94	1.96

Performance metrics for the 8 classifiers (Extra Trees Classifier, ETC; Gaussian Process Classifier, GPC; K-Nearest Neighbour, KNN; Logistic Regression, LOG; MultiLayer Perceptron Classifier, MLC; Ridge Classifier, RDC; Random Forest Classifier, RFC; and Support Vector Machine classifier, SVC; in descending order) used for the disambiguation in “Topic detection” for a random sample of 2000 genes. The metrics shown in this table were obtained by averaging the results on the validation set during the threefold cross validation. Subsequently, the results were averaged for a sample of 2000 genes. The logistic regression classifier (bold) was the fastest and second most accurate model for a random sample of 2000 genes and therefore it was selected as the default model to run the disambiguation on the remaining 17,082 human protein-coding genes. This high validation score verified that there was no over-fitting after the threefold cross-validation.

To test the performance of the disambiguation pipeline we compared the disambiguation results with the gene-publication annotations from GeneRif^58^ (manually curated annotations), DISEASES^59^ (computational annotations), and UniProt^60^ (computational and manually curated annotations) (Table 4). On average, the disambiguation recovers > 85% of all publications contained in these databases. Both GeneRif and Uniprot annotation do not necessarily contain a gene-synonym in the title or abstract, therefore those publications are out of our pipeline. Disambiguation results present on average a 70% precision with UniProt, the only collection of disambiguated publications of a similar magnitude. Finally, we included the disambiguated gene-publication annotations into the graph database.

**Table 4:** Comparison of disambiguation methods.

	Recall	Precision	Total annotations
Uniprot	0.86	0.71	10,329,240
DISEASES	0.90	0.14	1,140,129
GeneRIF	0.86	0.11	726,532
Ours	-	-	9,658,406

Average recall and precision of the disambiguation of our disambiguation with other databases. Low precision values for DISEASES and GeneRIFs are due to the smaller size of these databases.

### Trend detection

To detect incoming trends in the literature we gathered the publication dynamics of a given human gene from the disambiguated graph database (Fig. 1E). These time series include the number of publications, clinical trials, reviews and publications from big and medium-sized pharmaceutical companies, as well as, citations of publications coming from the mentioned categories per calendar year. Specifically, if a manuscript with author affiliations to big pharma cites other publications these citations are categorized as big pharma citations. Conversely publications citing this manuscript whose authors are affiliated to big pharma are not categorized as big pharma citations.

Time-series data from 1980 to 2013 was used to predict the per gene publication dynamics in each category between 2014 and 2019 using a Recurrent Neural Network model with an encoder-decoder architecture preceded by an attention layer, where both the encoder and decoder are composed of five hidden layers of Gated Recurrent Units (GRU). The time-series were created in a cumulative fashion, where each year contains the new publications and citations in addition to the previous ones.

**Figure 3:**
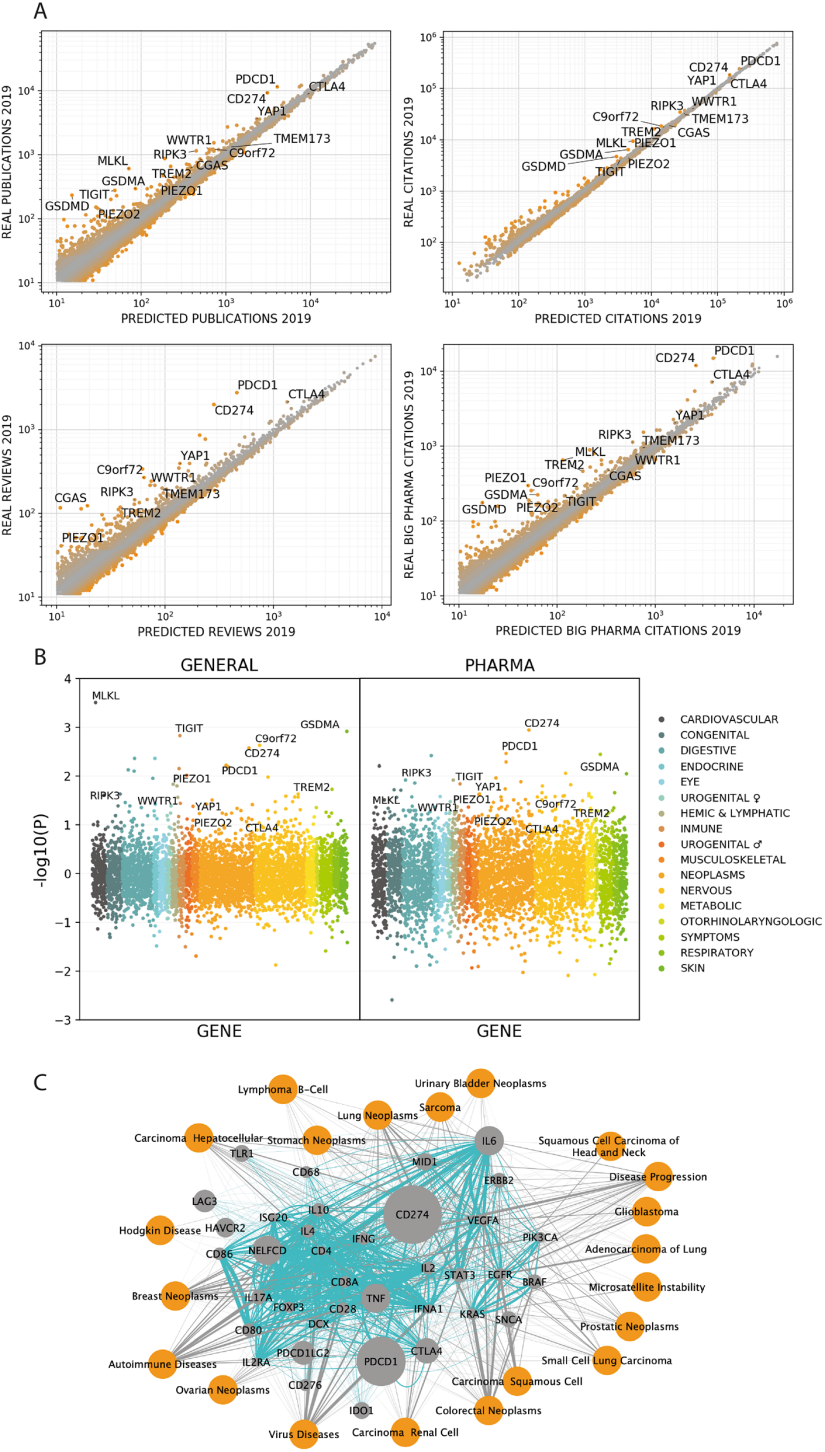
Trend detection and gene–gene-disease co-occurrence. **(A)** Logarithmic scatter plots showing the predicted number of publications, reviews, citations and citations from big pharma companies against real data in the year 2018. **(B)** Trendiness (log2(predicted/real)) for genes associated with groups of diseases (MeSH parent categories). Left; Average trendiness of publications, reviews, citations and citations from reviews. Right; Average trendiness of citations coming from big and medium sized pharmaceutical companies. **(C)** Gene–Gene–Disease co-occurrence network of the first neighbours of CD274. Orange nodes are diseases, grey nodes are genes and the size of gene nodes represents the trendiness The grey edges are gene-disease association, the blue edges are gene-diseases with the width of the edges reflecting the number of co-occurrences.

For most genes, the model produces accurate predictions of the publication dynamics (Table 5), but for a small subset of genes the real number of publications or citations is significantly higher than expected (Fig. 3A). When the number of publications or citations exceeds the predictions, we interpret that the publication dynamics changed substantially in a way that cannot be explained simply by the gene’s publication history, implying that a meaningful discovery in the field has recently occurred (Fig. 3A; orange). Trendiness is defined as the probability of the fold-change between predicted and real number of publications and citations for a given gene. We used this metric to identify the trendiest genes in the academic community-using all publications-, or in the pharmaceutical industry-using publications coming from pharmaceutical companies-(Table S1, supplementary material).

**Table 5:** Performance of the predictions.

Variables	MASE	Percentage of error	RMSE	Total (2013)
CIT. BIG PHARMA	0.42	12.51	10.60	1.86.E + 06
CIT. MED. PHARMA	0.50	14.90	5.20	6.59.E + 05
CIT. REVIEWS	0.30	3.35	45.60	2.38.E + 07
CIT. TRIALS	0.45	6.82	5.60	3.70.E + 06
CITATIONS	0.26	2.66	198.20	1.27.E + 08
PUB. BIG PHARMA	0.58	21.73	0.00	4.43.E + 05
PUB. MED. PHARMA	0.63	23.64	0.00	4.31.E + 05
PUBLICATIONS	0.33	8.66	32.60	9.48.E + 06
REVIEWS	0.52	13.37	2.40	9.07.E + 05
TRIALS	0.61	13.33	0.00	5.68.E + 05

Performance in the predictions of the publication dynamics. The model predicts the publications dynamics per gene between 2014 to 2019 using data from 1980 to 2013. Numbers represent the median 13,380 human genes.

*MASE* mean accuracy scaled error, *RMSE* root mean square error, *Total* number of elements in the database up to 2013.

Finally, to identify trendy genes of pharmaceutical interest, we computed the normalised mutual information of genes and diseases in the titles and abstracts of publications (Fig. 3B). Disease names and their synonyms were obtained from the Medical Subject Headings (MeSH) ontology at the Bioportal^61^. MeSH ontology contains 4818 different disease nodes at different levels of the ontology. We created a dictionary for each disease with the preferred and alternative names (see Methods). The diseases were disambiguated in titles and abstract using the same disambiguation pipeline used with the genes.

We noticed that many trendy genes cluster forming trendy pathways when getting the gene–gene and gene-disease association networks (Fig. 3C). We used enrichment of gene ontology (GO) terms for biological processes to uncover common pathways among the top 100 trendiest genes (Table S1, supplementary material). Among the most enriched GO terms in both academia and pharma are *T cell co-stimulation*, *execution phase of necroptosis* and *pyroptosis*. These biological processes are enriched in trendy genes which presumably reflect these fields of study are generating the most innovation and expectations in current biomedical research.

### Topic detection

**Figure 4:**
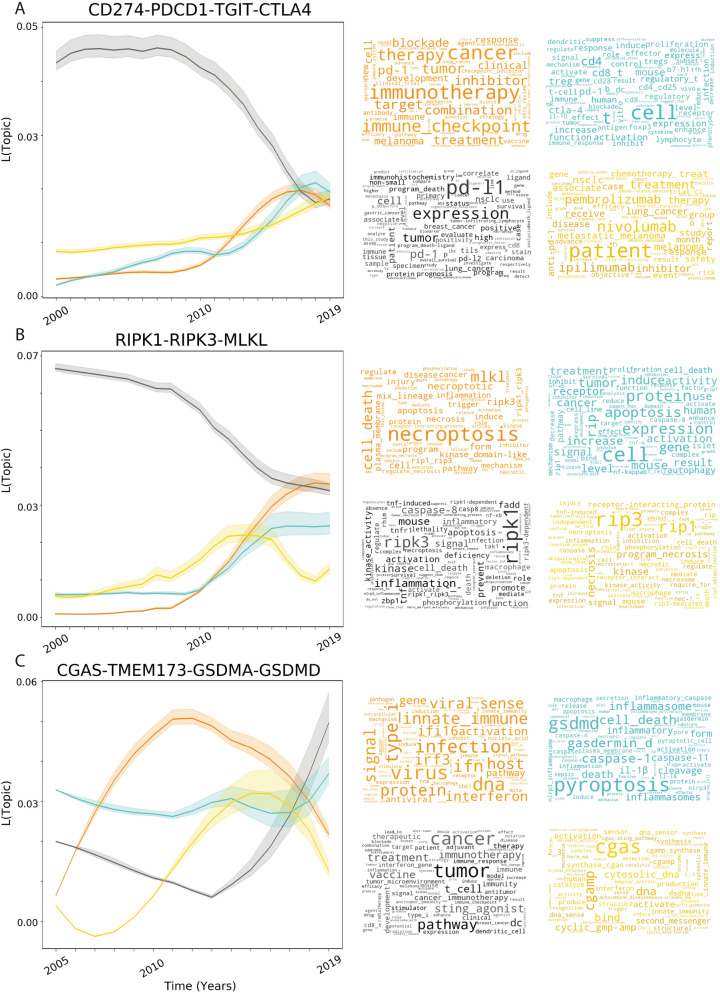
Topic time-lines. Topic time lines. Topic timelines for publications mentioning any of the genes for the immune checkpoint inhibitor **(A)**, necroptosis **(B)** and pyroptosis **(C)** pathways. The x-axis represents the time in years and the y-axis represents the likelihood of a given topic. Colors represent different topics defined by the keywords contained in the correspondent word clouds. The latent four topics were obtained using Non-Negative-Factorization all publications annotated with the genes after disambiguation. Word clouds were created using the phrases with highest TFIDF for groups of publications belonging to each topic. All timelines show at least one rising topic after 2013 that represents the reason why these genes became trendy, their implications in human disease: immune checkpoint inhibitors and monoclonal antibodies (yellow and orange in **A**), activation of necroptosis (orange in **B**), agonists of STING1 in cancer (black in **C**).

After the detection of gene trends, the next step was to understand why those genes might be trendy and curate possible mistakes in the disambiguation. With this aim we implemented a topic detection pipeline as an automatic, fast discovery tool to study groups of publications that mention the gene of interests (Fig. 1F). In this context, we used topic modelling algorithms. A topic is a collection of similar words, specific to a group of documents^62^. We used non-Negative Matrix Factorisation to generate a set of latent topics for each query (Fig. 4A; word clouds).

We explored the evolution of the topics associated with some trendiest genes. For the immune checkpoint inhibitors (CD274, PDCD1, TGIT and CTLA4) the topic timeline suggests that there was a rapid decrease in the likelihood of publications discussing the biological role of these immune checkpoint inhibitors since 2010 (Fig. 4A in grey), which coincides with a notable increase in topics that discuss cancer therapies (Fig. 4A in orange) and monoclonal antibodies that target these four different transmembrane immunoglobulins (Fig. 4A in yellow).This way, the topic-detection pipeline is able to capture the evolution of the research from its biological description to the clinical application.

The topic timeline of the members of the necroptosis pathway (RIPK1, RIPK3 and MLKL; Fig. 4B) suggests that in the last decade there has been a decrease in the likelihood of publications discussing these genes in the context of apoptosis (Fig. 4B in grey), in favour of publications that discuss the newly discovered form of cell death, the necroptotic pathway (Fig. 4B in orange), as well as, the translational medicine perspective of this pathway as is suggested by words like mouse, treatment and activity or cancer (Fig. 4B, in blue).

Finally, the topic timeline the members of the pyroptosis pathway (CGAS, TMEM173, GSDMA and GSDMAD; Fig. 4C) shows a fast increase from 2013 of publications discussing the therapeutic opportunity in cancer immunotherapy with agonists for TMEM173 (Fig. 4B in grey), while again, the remaining topics seemed to contain information on the biochemistry and biological role of the genes.

### Recommender system

In addition to the automatic topic detection, we designed a review recommender system to accelerate the screening of the publications that cover most of the information in a network (Fig. 1G). There are an average of 2.9 reviews citing any publication that mentions at least one gene name. The aim was to minimise the time reading and maximising the information within a gene subnetwork. The algorithm aggregates both topic and network information from the citation subgraph of the publications that mention the gene of interest to obtain the most query-centric reviews. The topic information comes from the latent topics obtained from the topic detection algorithm. The network information was captured by the PageRank scores of the subgraph (see Methods). This approach ensures that reviews citing publications with highest PageRank scores are prioritised. To further minimize the number of reviews for initial human analysis we avoid repetition of information by simultaneously maximising the cumulative PageRank score whilst minimising the overlap of combined citations. This way, we expect to obtain a small set of reviews that will cover the main topics and publications in the field. We used this recommender system to select the optimal subset of reviews to assess why genes might be trendy (see Discussion). An example of the output can be found for the genes in the discussion in the supplementary data file.

## Discussion and conclusions

We present TrendyGenes as a first attempt to (i) establish a systematic analysis of contemporary topics associated to human genes and diseases, (ii) develop an alert system for emerging targets and trends in the scientific literature across the human, protein-coding genome, (iii) to use topic modelling to rapidly generate timelines of phrases that facilitate the understanding of why these genes are trendy.

We constructed a graph database containing PubMed data where publications are connected by citations and authors and are annotated with disambiguated human gene-names and diseases. We expect this new resource to provide new ways to navigate the scientific literature, detect and visualise networks of discussion and analyse networks of influence from key opinion leaders. Disambiguating author names from PubMed, MedRxiv, or BioRxiv would further improve the quality of the database. Machine or deep learning algorithms could be trained on already labelled data to improve on previously published approaches^63–65^ and address this issue.

Similarly further improvements in gene-name disambiguation would assist precision and recall metrics on our validation set suffer for different reasons GeneRIF and DISEASES include fewer publications in comparison to the genome wide metrics identified in our pipeline and there will be a lot of potential “false positives”. This makes the precision of our approach appear lower than what it may actually be. On the other hand, GeneRIF and Uniprot contain publications which either are not gene specific or do not mention the gene in the title or abstract.

However, the disambiguated genes and diseases can serve as labelled data for more sophisticated deep learning approaches to annotate biomedical entities. Gene and disease entities could be better annotated using both representation learning to capture the network topology and contextual information with transformer layers. Topic detection could be improved by using the state-of-the-art text summarisers with deep learning.

The number of publications per gene in aggregate is very predictable^66^. However, occasionally genes present significantly more publications than expected, meaning that a recent breakthrough occurred which cannot be accounted for from the publication dynamics. In this study, we show that *trendiness* can identify emerging targets from the literature for rapid profiling at genome-scale. We combined trendiness with gene-disease associations to prioritise potential drug targets: emergent genes associated with diseases but yet included in pharmaceutical publications are worthy of being investigated as potential targets. We observe that TrendyGenes usually cluster into the same biological pathways (Fig. 3C for CD274, PDCD1, CTLA4 and TIGIT). Here, using topic modelling and the recommendation system, we identify the trendiest genes and pathways and discuss some case studies to exemplify our pipeline. We selected genes pharmacological relevance by choosing genes with high trendiness both in the academia and the pharmaceutical industry with high association with disease and more than 100 publications. Reviews suggested by the recommender system for these genes are included in (whatReview2read.zip, supplementary material).

### Immune checkpoint inhibitors: CTLA4, CD274, PDCD1, TIGIT

CTLA4, PDCD1 (PD-1), CD274 (PD-L1) and TIGIT are among the trendiest genes in academia and pharma in 2019 (Fig. 3A). CTLA4, PDCD1, CD274 and TIGIT genes encode four different transmembrane immunoglobulins that act as co-inhibitory receptors: checkpoints or ‘breaks’ for the adaptive immune response that prevent T cells from exerting their functions^67,68^. CTLA4 competes with its analogous CD28 for CD80 and CD86 to prevent a premature activation of T cells^68^. PDCD1-CD274 interaction counters the positive signals that may have already activated T effector cells^68^. TIGIT interacts with CD155 to down-regulate natural killer cells and T lymphocytes^69^. Cancer cells attempt to impair these checkpoints and currently there are 7 FDA approved monoclonal antibodies that target three of proteins (CTLA4: Ipilimumab^70^; PDCD1: Nivolumab^71^, Pembrolizumab^72^, Cemiplimab^73^; CD274: Atezolizumab^74^, Avelumab^75^) and multiple candidates targeting TIGIT (BGB-A1217^76^, OMP-313M32^77^, MTIG7192A^78^, AB154^79^). Moreover, James Allison and Tasuku Honjo received the Nobel Prize in Medicine in 2018 for its research on immune checkpoint inhibitors^47^.

### Neurodegeneration: TREM2 and C9orf72

C9orf72 encodes a guanine nucleotide exchange factor involved in endosomal trafficking and autophagy^80,81^. Hexanucleotide repeat expansions in promoter or intronic regions of C9orf72 are some of the major causes of sporadic and familial forms of both amyotrophic lateral sclerosis and frontotemporal dementia^80^. Antisense oligonucleotides are being used to impede the transcription of C9orf72^82–84^ or CRISPR–Cas9 system to target the GGGGCC repeat in the DNA^85^ or RNA^85,86^.

TREM2 gene encodes a transmembrane immunoglobulin receptor expressed in macrophages, osteoclasts, dendritic cells, and brain microglia^87,88^. TREM2 variants have been associated with Nasu-Hakola disease^89,90^, late-onset Alzheimer’s disease^91–94^, frontotemporal dementia^95–100^, amyotrophic lateral sclerosis^101,102^ and Parkinson’s disease^101,103^. TREM2 activates a pathway—through TYROBP/DAP12—that promotes inflammation^87,88^ and promotes phagocytosis of cellular waste, remains of apoptotic cells, and pathogens^87,88^. Currently, two independent groups have generated anti-TREM2 antibodies to stimulate microglia to remove amyloid plaques^104^. Furthermore, the mAb generated by one of these groups, Alenco, in collaboration with Abbvie, has entered Phase I clinical trials^105,106^.

### DNA sensing by cGAS–STING: cGAS, TMEM173, GSDMD, GSDMA

The cytosolic nucleic acid-sensing pathway leads to pyroptosis, a lytic pro-inflammatory type of cell death involved in antiviral, antibacterial, and anticancer response^107^. cGAS is a nucleotidyl-transferase that catalyzes production of cyclic GMP-AMP (cGAMP) upon the recognition of double-stranded DNA^107^. TMEM173 (STING) binds to cGAMP and promotes the activation of both TBK1 and IRF3, increasing the transcription of genes encoding type I interferons^107^. GSDMA and GSDMD are pore-forming effector proteins in the plasma membrane to release proinflammatory interleukins like IL-1β and IL-18^108^. The cGAS-STING pathway has been associated to multiple autoimmune and chronic inflammatory diseases like non-alcoholic fatty liver disease^109^, systemic lupus erythematosus^110^, vascular and pulmonary syndrome^111^, macular degeneration^112^, Bloom syndrome^113^, Aicardi-Goutières syndrome^114^, cancer^115^, DNA damage^116^, neurodegeneration^117^ and beyond. Currently, there are ongoing clinical trials for TMEM173^118–120^ and GSDMD^121^ although there are no reported trials for GSDMA nor cGAS.

### Necroptosis: RIPK1, RIPK3, and MLKL

RIPK1, RIPK3 and MLKL form part of the tumour necrosis factor-induced necroptosis pathway^122–124^. This pathway has been associated with multiple pathologies: systemic inflammatory response syndrome^125,126^, ulcerative colitis^127,128^, psoriasis^128^, rheumatoid arthritis^128^, neurodegenerative diseases^129^ and even cancer^130–132^. TNFR1, FasL, TRAIL, and TLR can all activate RIPK1 to decide the cell’s fate: inflammation, apoptosis or necrosis^133^. If caspase-8 is inhibited, RIPK1 and RIPK3 form the necrosome that subsequently phosphorylates MLKL^134^. MLKL forms homo-trimers^135,136^, migrates to the plasma membrane^135,136^, binds to highly phosphorylated inositol phosphates^137^, creates pores in the membrane^138^ and disrupts the cell integrity. The discovery of RIPK1 dates back to 1995^139^. Since then, four inhibitor programs have progressed through human phase II safety trials^140–143^. The first publication mentioning MLKL is more recent^144^ and, despite the lack of kinase activity, pharmaceutical companies have cited its publications by 60 times more since 2013. Although there are no clinical trials yet, there are at least three known different chemical inhibitors^145^.

### Mechanobiology: YAP1/WWTR1, PIEZO1 and PIEZO2

Cells use mechanical cues from their environment to guide behaviours such as proliferation and migration. Forces act as signals which are transduced to the nucleus where they control gene expression^146^. Mechanical forces are critical regulators of organ and tissue homeostasis, morphogenesis and regeneration, and are important aspects of diseases like cancer, metastasis, fibrosis and cardiac hypertrophy. YAP1/WWTR1 (TAZ) are transcriptional co-activators and mechanotransducers^147^. YAP/TAZ is hyperactivated in cancers^148^, its inhibition reduces atherogenesis^149^ and fibrosis^150^, it triggers pulmonary hypertension^151^ , and it is necessary for epithelial regeneration in the intestine^152^. PIEZO1 and PIEZO2 are two mechano-sensitive cation channels that play a key role in cell number regulation^153,154^ and migration^155^, hearing^156^, neural^157^ and vascular^158^ development, somatosensory functions^159^, proprioception^160^ and beyond. Piezo channels have been recently associated with multiple pathologies like arthrogryposis^161^, apnea^162^, congenital lymphatic dysplasia^163^, hyperalgesia^164,165^, malaria^166^, pancreatitis^167^, xerocytosis^168^, Gordon syndrome, Marden-Walker Syndrome, and Distal Arthrogryposis Type 5^169^. The discovery of mechanotransduction signalling pathways has received notable attention in the last years and may open the door to new therapeutic strategies to treat these diseases^147^.

Trends in scientific literature are useful for pharmaceutical and biomedical companies. Moreover, this approach can offer crucial information to funding agencies to prioritise projects and a new way to study the research impact. Finally, individual researchers may benefit from a new methodology to explore the literature and from algorithms to maximise the efficiency of navigating over an increasingly vast biomedical literature.

## Material and methods

### Terminology

Here we use the term gene symbol to mean the approved symbol for any of the 19,084 human, protein-coding genes accepted by the HUGO Gene Nomenclature Committee. We refer to gene synonyms as any of the possible gene name variations by which the scientific community has ever referred to a given gene. Approved gene symbols are also included in the gene synonyms. For example: ‘EGFR’ is the approved gene symbol whereas ‘EGFR’, ‘Epidermal Growth Factor Receptor’, ‘ERBB1’, ‘ErbB-1’, ‘c-erbB1’, ‘HER1’, ‘ERBB’ are gene synonyms. We define promiscuous gene names as any gene synonym that is a synonym of more than one gene. This can include previous official gene symbols since these will not have been expunged from the literature. An example of this could be ‘ARP1’ which is a promiscuous gene synonym for the gene symbols ‘NR2F2’, ‘ACTR1A’, ‘ACTR1B’, ‘ANGPTL1’, ‘APOBEC2’, ‘ARFRP1’, ‘PITX2’^47^. Unsafe gene synonyms are gene synonyms that may have a different meaning in other areas of research or in another context, for instance in standard English. The ‘STAR’ gene symbol is unsafe as opposed to its gene synonym ‘Steroidogenic Acute Regulatory Protein’ or CCP4 is both a gene synonym and the name for crystallography software. The final type of synonym we distinguish are Nested gene synonyms. These are gene synonyms that are part of another gene synonym. For instance ‘insulin’ is a nested gene synonym of ‘insulin receptor’, ‘TNF’ is nested gene synonym of ‘TNF Receptor Superfamily Member 1A’ (gene symbol ‘TNFRSF1A’) and ‘TNF Receptor Associated Factor 2’ (gene symbol ‘TRAF2’).

### Pubmed as a graph database

PubMed baseline 2020^53^ comprises 30,419,056 publications for biomedical literature from MEDLINE and life science journals and 173,572,773 citations from the full-text archive of open-source publications PubMed Central (PMC). PubMed was imported into a graph database (Fig. 1A) for a fast performance in the retrieval of highly relational data like authorship and citation networks. In a graph database information is represented as nodes and edges, allowing the fast retrieval of queries about relationships. We loaded PubMed 2020 base-line into Neo4J^54^, an open source graph-database management system. We introduced four node types (publications, authors, human protein-coding genes, human diseases, medical subheadings), and four edge types (*published*, from authors to publications; *cited by* between publications, *gene annotation* from genes to publications; and *disease annotation* from diseases to publications). Furthermore, PUBLICATION nodes have the following attributes: PubMed identifier, title, abstract, affiliations, is_review, is_clinical_trial, big_pharma, med_pharma and date of publication. Profiling of the graph is included in Table 6, Database Profiling. Neo4J offers an interactive approach to navigate through PubMed (i) easily accessing references of publications, (ii) with the ability to query for specific genes and diseases already disambiguated, and (iii) with the aim of creating a knowledge graph for further exploration of gene-disease associations. The database is accessible to download at: https://zenodo.org/record/8362679.

**Table 6:** Database profiling.

Graph entity	Type	Counts
PUBLICATION	Node	30,419,056
AUTHOR	Node	8,331,251
GENE	Node	19,082
DISEASE	Node	4818
MESH	Node	29,133
CITED_BY	Relationship	173,572,773
PUBLISHED	Relationship	121,879,576
GENE_PMID	Relationship	9,656,712
DISEASE_PMID	Relationship	39,605,276
MESH_PMID	Relationship	279,331,447

We loaded PubMed 2019 base-line into Neo4J, an open source graph-database management system. We introduced four node types (PUBLICATION, AUTHOR, GENE, DISEASE), and four edge types (PUBLISHED, from AUTHOR to PUBLICATION; CITED_BY between PUBLICATION, GENE_PMID_ASSOCIATION from GENE to PUBLICATION; and DISEASE_PMID_ASSOCIATION from DISEASE to PUBLICATION). Furthermore, PUBLICATION nodes have the following attributes: PMID, TITLE, ABSTRACT, AFFILIATIONS, IS_REVIEW, IS_CLINICAL_TRIAL, BIG_PHARMA, MEDIUM_PHARMA and DATE. The database is accessible at: https://mega.nz/file/4E8QjCaQ#oqtm7jof-lsG7ySget8uakh7m26bDLo1HrPu3mtdAV8.

### Gold standard sets

GeneRif^58^, UniProt, and DISEASES^59^ were used as a golden-standard for validation.

### Pharmaceutical companies

A list of organisation names was generated from Cortellis^170^. Organisations with more than 100 patents in Cortellis were considered ‘big pharma’ and ‘mid pharma’ otherwise.

### Gene annotation

#### Search for publications

A ElasticSearch API search engine was used to retrieve PubMed IDs of publications containing a gene or disease synonym in their title, abstract or keywords (Fig. 1D; GET PMIDs and GET corpus). These PubMed IDs were later used to retrieve the publications’ attributes from Neo4J using Cypher language through its python driver^171^ (Fig. 1E; GET trends). Regular expressions were used avoid nested name ambiguity with lookarounds and fuzzy matching to account for case and punctuation and letter case variations (e.g. ‘ErbB-1’, ‘erbB1’, ‘ERBB1’, ‘ErbB 1’).

#### Unsafe synonym detection

We used 19,082 protein-coding human genes annotated by HUGO Gene Nomenclature Committee (HGNC). Gene synonyms were obtained from Ensembl, HUGO, Entrez, UniProt and Open Targets (Fig. 1C). Gene synonyms which were identical to disease names contained in the Medical Subject Headings (MeSH) database were eliminated. This mainly occurs when genes are named after diseases that are associated with e.g. ‘Li Fraumeni syndrome’ as a gene synonym for gene TP53^172^ or ‘Marfan syndrome’ in ‘FBN1’^173^.

Gene synonyms were classified into “safe” or “unsafe” categories using a modified version of the positive-unlabelled learning with bootstrap-aggregating as implemented by Mordelet et Vert (PU-learning; Fig. 1C)^55^. PU learning is a form of semi-supervised learning which iteratively finds positive examples within a-priori unlabeled data. To build a binary classifier able to distinguish the unlabelled class (U) into unsafe (P, positive) and safe (N, Negative) we engineered a series of features (Table 2, Unsafe features) such as the combined frequency of the characters in a gene synonym (example: ‘ZNF’ will be safer than ‘EDA’ because ‘Z’ and ‘F’ characters are less frequent in PubMed corpus than ‘E’, ‘D’, and ‘A’) or the probability of a gene synonym given that other gene synonym appeared in the text (the probability of ‘STAR’ given ‘Steroidogenic Acute Regulatory Protein’ is high but the probability of ‘Steroidogenic Acute Regulatory Protein’ given ‘STAR’ is low because ‘STAR’ is more ambiguous).

The PU learning was run for five iterations with a random forest classifier. The pure positive class (unsafe) was constructed combining gene synonyms present in the Enchant English dictionary^174^, gene synonyms with less than three characters, and promiscuous gene synonyms. In an active learning fashion, after each iteration, the top 1000 examples with the highest probability of being unsafe were manually relabelled if they were wrongly classified. For example, true positive unsafe synonyms like gene families (e.g. ‘G protein coupled receptor’), phenotypes (e.g. ‘Williams Beuren Syndrome’) and other biological entities (e.g. ‘Cell surface antigen’) were included in the true positive set for the next iteration. False positives like ‘thymopoietin’ or ‘tubulin alpha-1C chain’ were included into a new true negative class for the remaining iterations.

After the five iterations, a gene synonym was considered unsafe if: (i) it is included in the English dictionary from Enchant, (ii) it is a word with less than three characters, (iii) if the predicted score for the random forest classifier was higher than 0.5, and (iv) it is a promiscuous gene synonym .

#### Community detection

We produced weighted, undirected co-citation networks from unweighted, directed citation networks (Fig. 1D, GET communities). Subsequently, connected components were broken into communities using the fast greedy modulation algorithm implementation in iGraph^175^.

#### Gene annotation

Communities in co-citation networks represent different areas in the scientific literature. We used this feature to disambiguate large groups of publications (Fig. 1D, LABEL communities). We labelled all the publications in a community with the gene symbol of interest if the ratio of publications mentioning at least one safe synonym with respect of publications that only mention unsafe synonyms was higher than 0.1%.

Nevertheless, the co-citation graph is incomplete because PMC only contains citations of open-source publications. Because of that, 46% of the publications were disconnected from the co-citation graph. Disconnected publications that mention a safe-synonym were automatically linked to the gene symbol of interest. The rest of disconnected publications were linked to gene of interest using PU bagging strategy^55^ with a binary logistic regression classifier based on the words in the text corpus (keywords, titles and abstracts) of communities already linked to the gene of interest and discarded communities.

Each corpus was pre-processed by (i) removal of non-alphanumeric characters, (ii) tokenization or split by whitespace, (iii) deletion of stop words from NLTK^176^, (iv) lower case conversion, (v) deletion of tokens whose length is less than three characters, (vi) deletion of token representing integers and (vii) stemming (‘disambiguated’, ‘disambiguations’, ‘disambiguating’ is converted to ‘disambiguat’). List of tokens (uni-, bi-, tri-, tetra-grams) with at least 2 counts and a frequency lower than 0.6 in the complete corpus were vectorised using TF IDF^177^. When there were less than 1000 unlabeled publications in the training set for the gene of interest, we generated an auxiliary negative class to augment the negative examples in the training data. This auxiliary negative class comprised a random sample of 1000 publications that mentioned genes different from the gene of interest.

These vectors were fed to all available machine learning classifiers from the Python library sklearn: Extra Tree Classifier, Gaussian Process Classifier, K-Nearest Neighbour, Logistic Regression, Ridge Classifier, Random Forest Classifier, and Support Vector Machine. All classifiers were trained with hyper-parameter tuning and threefold cross-validation to avoid over-fitting in each of the 50 PU-bagging iterations (Table 7). The implementation of this PU learning algorithm is the same as the inductive bagging positive-unlabelled learning with bootstrap-aggregating approach described by Mordelet^55^ (PU-learning; Fig. 1D) and also the same as in the section of “Unsafe Synonym Detection” of the Methods (PU-learning; Fig. 1C). To maximise specificity and sensitivity simultaneously we selected the models with highest weighted F1 score (Scikit Learn) to maximise precision and recall at the same time. We selected the logistic regression (LOG, Table 3) classifier for the disambiguation pipeline given its accuracy-speed balance (Table 3).

**Table 7:** Hyper-parameters for model selection.

ClassifierName	HParamName	ValuesToUse
ExtraTreesClassifier (ETC) / Random Forest Classifier (RFC)	bootstrap	[0, 1]
	class_weight	["balanced"]
	criterion	["gini","entropy"]
	max_depth	[10, 20, 30]
	max_features	[“auto”, “sqrt”, “log2”, 0.5]
	max_samples	[None, 0.6]
	min_impurity_decrease	[1e−5, 1e−4, 1e−3]
	min_samples_leaf	[2, 6, 10, 20]
	n_estimators	[100, 200]
	oob_score	[0, 1]
	random_state	[321]
GaussianProcessClassifier (GPC)	kernel	[RationalQuadratic, RBF]
	n_restarts_optimizer	[0, 1, 2]
	random_state	[321]
KNeighborsClassifier (KNC)	algorithm	["ball_tree", "kd_tree"]
	leaf_size	[10, 20, 30, 40, 50]
	metric	["euclidean","minkowski","mahalanobis","chebyshev"]
	n_neighbors	[2, 5, 10, 15]
	random_state	[321]
MLPClassifier (MLC)	activation	["sigmoid","relu","tanh"]
	alpha	[1e−3, 1e−4, 1e−5]
	early_stopping	[True]
	epsilon	[1e−6, 1e−8]
	hidden_layer_sizes	[(10,),(50,),(100,),(10,10,),(50,50,),(100,100,),(10,10,10,),(50,50,50,),(100,100,100,)]
	learning_rate	["adaptative"]
	learning_rate_init	[1e−3, 1e−2, 2e−2]
	n_iter_no_change	[2]
	random_state	[321]
	solver	["adam"]
	validation_fraction	[0.1]
RidgeClassifier. (RDC)/LogisticRegression (LOG)	alpha	[321]
	class_weight	["balanced"]
	fit_intercept	[True]
	max_iter	[2000]
	random_state	[321]
	solver	[‘lsqr’ (RDG), ‘sparse_cg’ (RDC), ‘sag’, ‘saga’, ‘lbfgs’ (LOG), ‘liblinear’(LOG), ‘newton-cg’(LOG)]
	tol	[1e−3, 1e−4, 1e−5, 1e−6]
SVC	class_weight	["balanced"]
	fit_intercept	[True]
	C	[0.1, 1, 10]
	degree	[1, 2, 3, 4]
	kernel	[‘linear’, ‘poly’, ‘rbf’, ‘sigmoid’]
	random_state	[321]

Hyper-parameters used during the model selection for Table 3. ClassifierName column contains the name of the classifier and our acronym. Some classifiers were grouped since they have similar hyper-parameters like ExtraTreesClassifier (ETC) and Random Forest Classifier (RFC); and RidgeClassifier (RDC) and LogisticRegression (LOG). HParamName contains the names of the hyper-parameter names in the same format as sklearn version 0.24.2 (stable). ValuesToUse column contains the list of potential values of those hyper-parameters to be evaluated. Some values are specific for only one classifier and therefore have the acronym for the model in parenthesis (e.g. ‘lsqr’ (RDC) and ‘lbfgs’ (LOG)). random_state and class_weight hyper-parameters were intended to have the same value across all models. The validation_fraction was used in MLPC to use the feature of early stopping : this created a sub-validation set under the training set different from the validation sets created for the cross-validation.

### Disease annotation

The same procedure used for gene entity recognition was used to detect disease entities, co-citation networks and machine learning. The Medical Subject Headings (MeSH) ontology was downloaded by querying their Rest-API available at BioOntology^178^. We constructed a key-value dictionary. Each disease was a node in the ontology. The disease synonyms were obtained from the 'Concept List Terms' field in the ontology to gather the preferred and the alternative ways of denoting the disease. We generated more synonyms of the diseases by reversing the order of synonyms with commas: 'Insipidus, Diabetes' to 'Diabetes Insipidus'.

### Gene–gene-disease co-occurrence

#### Co-occurrence

Co-occurrence of genes and diseases was computed using the simultaneous occurrence of gene/disease tags in publications after disambiguation, normalized by the total number of publications presenting those tags. We also computed mutual information metrics for gene–gene and gene-disease associations.

#### Gene mesh-parent association

Every disease MeSH term was associated with its lowest ancestor in the MeSH ontology under the node Disease^179^. After computing the gene-disease co-occurrence, each gene is linked with the most frequent ancestor disease term (Fig. 3B).

### TrendyGenes

#### Trendiness

In this paper the trendiness for a gene is defined as the probability of observing the magnitude of fold-change between the predicted and the real number of publications for that given gene. The error in the predictions is inevitably higher with genes associated with small numbers of publications. To correct for this, we generated five bins based on the initial number of publications (percentiles 20, 40, 60, 80 and 100). We computed the distribution of the fold-changes between the predictions and observed reality in each of the five bins using a gaussian kernel density estimator available at Scikit Learn (bandwidth = 0.1, remaining parameters with default values). The area under the obtained probability density function is equal to 1. Trendiness is the area of the right tail of the probability density function bounded to the left by the observed fold change. This gives us an estimate of how extreme the fold change was for that gene in a specific bin.

#### RNN model

The model consists of an encoder-decoder architecture preceded by an attention layer. Both the encoder and decoder were composed of five hidden layers of Gated Recurrent Units (GRU). The model was implemented in Keras using the Tensorflow-GPU backend. Min–max normalization was used to rescale the time series before training. The optimizer was RMSprop and the loss was computed as the log error. 30 percent of the time series was reserved for validation during the training.

#### Model optimisation

Input data was in both forms: cumulative and differential. In the cumulative model each year contains all the publication and citations published until then, while the differential model only contains the publications published in that year. Multiple normalisations were used ('none', ‘minmax’, ‘log’, ‘standard’, and combinations of them). Similar results were obtained with different normalisations and minmax was finally selected. Multiple Recurrent Neural Networks (RNNs) architectures were used (GRU, LSTM) in the form of encoder-decoder, with different numbers of neurons (1, 5, 10, 20, 50; Table 8). Models were compared with the Mean Accuracy Scaled Error (MASE), an unbiased method to compare time-series prediction models by comparing how much each model out-performs a naive model that repeats the last value. The 5-neuron-GRU with cumulative time-series was selected because it was the most parsimonious model with the smallest MASE.

**Table 8:** Model size comparison.

Variables	Median MASE				
	1 GRU	5 GRU	10 GRU	20 GRU	50 GRU
CIT. BIG PHARMA	0.46	0.42	0.42	0.41	0.42
CIT. MED. PHARMA	0.51	0.50	0.47	0.48	0.48
CIT. REVIEWS	0.38	0.30	0.25	0.24	0.26
CIT. TRIALS	0.45	0.45	0.43	0.43	0.43
CITATIONS	0.39	0.26	0.21	0.23	0.25
PUB. BIG PHARMA	0.66	0.58	0.56	0.54	0.55
PUB. MED. PHARMA	0.76	0.63	0.56	0.55	0.55
PUBLICATIONS	0.48	0.33	0.26	0.26	0.26
REVIEWS	0.53	0.52	0.50	0.46	0.50

Global performance of the RNN model. Mean accuracy scaled error (MASE) is an unbiased method to compare time-series prediction models by comparing how much each model out-performs a naive model that repeats the last value. Values below 0 indicate that the method performs better than the naive model. Data for 13,380 human genes.

#### Gene ontology terms enrichment

For the enrichment of gene ontology terms (Biological Process) associated with the 100 trendiest genes in academia (all publications) and the pharmaceutical industry we used the online tool GeneCodis 4.0^180^ with default parameters.

### Recommender system

#### Topic detection

We implemented algorithms to detect topics in collections of publications. This is useful to determine in which areas trendy genes are relevant. Furthermore, topic detection allows the fast identification of errors during the disambiguation. We used two different topic detection algorithms: Latent Dirichlet Allocation^62^ (LDA), and Non-Negative Matrix Factorisation^181^ (NMF). Both algorithms factor a nonnegative matrix ‘A’ with size NxM, where N is the number of publications and M is the dimension of the TF IDF vector obtained for Named Entity Recognition (see above), into non-negative factors matrix W of size NxK and matrix H with size KxM where WxH is an approximation of matrix A. The matrix W contains the strength of the association of a given publication to belong to a latent topic while H contains the strength of the association between a latent topic and a given n-gram. Scikit Learn implementations for both algorithms were used to generate 'K' number of topics defined by the user with the default parameters until convergence (tolerance of 1e−12). We previously used perplexity^182^ to select the optimal number of topics but we disagreed with the output: the number of topics that model the corpus better was not necessarily the most human interpretable. Topic timelines were obtained by calculating the mean and standard deviations of the topic probabilities for all publications mentioning the gene of interest per calendar year (Fig. 4).

#### Recommendation algorithm

We implemented an automatic pipeline to guide users about which reviews to read for a specific query in PubMed. To do that, we used the topic probability of the publications and an aggregated PageRank score of the citation networks. The user can select an interval number of reviews (R) that is willing to read: between 2–3 or 3–50. Then, three matrices are defined for each group of publications: (i) a binary, sparse matrix of size NxR with N publications and R reviews that comprised the citation adjacency network; (ii) a Nx1 weight matrix that comprise a PageRank scores; and (iii) a NxK matrix with the topic probabilities for N publications and K user-defined topics.

The score for each review was defined as the sum of the PageRank scores of its references while the score for a combination of reviews is defined as the row sum of the indexed NxR matrix multiplied by the Nx1 PageRank vector and the sum of the obtained vector. Results were later normalized by the total maximum score, defined as a hypothetical review citing all gene publications. Finally, every combination of R reviews is presented to the user with the score, the average of the cited publication dates and the probability to belong to one of the K previously defined topics.

### Supplementary Information

Supplementary Dataset.

Supplementary Table S1.
